# Ecological and Socioeconomic Factors in the Occurrence of Rabies: A Forgotten Scenario

**DOI:** 10.3390/idr14060097

**Published:** 2022-12-01

**Authors:** Deepak Subedi, Deepak Chandran, Sanju Subedi, Krishna Prasad Acharya

**Affiliations:** 1 Paklihawa Campus, Institute of Agriculture and Animal Science (IAAS), Tribhuvan University (TU), Siddarthanagar 32900, Nepal; 2 Department of Poultry Science, University of Georgia, Athens, GA 30602, USA; 3 Department of Veterinary Sciences and Animal Husbandry, Amrita School of Agricultural Sciences, Amrita Vishwa Vidyapeetham University, Coimbatore 642109, Tamil Nadu, India; 4 School of Public Health, Chitwan Medical College, Tribhuvan University (TU), Bharatpur, Chitwan 44200, Nepal; 5 Animal Quarantine Office (AQO), Department of Livestock Services (DLS), Kathmandu 44600, Nepal

**Keywords:** rabies, vaccination, post-exposure prophylaxis, ecological factor, socioeconomic factor

## Abstract

In many third world countries, where rabies is endemic in dog populations, humans continue to be at risk of contracting the disease. Vaccination is the most effective form of prophylaxis for people, yet it often fails to adequately protect dogs. The most major implications are the costs of post-exposure prophylaxis (PEP) after an exposure occurs and the loss of human life and productivity due to early mortality from rabies (about 60,000 deaths annually). The largest rabies death tolls can be found in the world’s poorest regions, where rabies vaccinations for domestic dogs are uncommon and PEP is scarce. Mass vaccination of dogs, neutering programs, patient PEP, strengthening laboratory and human resources, education and awareness, and animal and human rabies surveillance are all common methods used to prevent, control, and ultimately eradicate dog-mediated human rabies. Current rabies control initiatives, however, pay little attention to the role that ecological and socioeconomic variables play in the disease’s occurrence and spread. To help better inform rabies control strategies, we address in this work the ways in which ecological and socioeconomic factors affect the occurrence and spread of rabies.

## 1. Introduction

Rabies is a viral (lyssavirus), 100% fatal, and vaccine-preventable disease that can infect all mammals. In rabies-endemic countries, nearly 99% of the cases in humans are caused by dog bites [1,2]. The World Health Organization (WHO) estimates that between 59,000 and 60,000 human deaths are caused by rabies each year, with South Asian countries responsible for approximately 45% of these deaths [1,3]. Around 59% of global rabies deaths are recorded in Asia, followed by 36% in Africa; the majority of these nations fall into the category of low- and middle-income countries (LMICs). The remaining 5% of human deaths occur in other LMICs, with relatively few cases reported from high-income countries (HICs) [4,5,6,7]. Common strategies for preventing, controlling, and ultimately eliminating dog-mediated human rabies include mass dog vaccination, neutering programs, post-exposure prophylaxis (PEP) of patients, strengthening of laboratory and human resources, education and awareness, and animal and human rabies surveillance [7,8,9]. However, the social and economic factors that come into play during the occurrence and transmission of rabies are largely neglected in present rabies control programs. Thus, the current paper explore the ways in which environmental and social factors contribute to the spread of rabies, providing crucial context for rabies prevention efforts. 

## 2. Ecological Factors

Several ecological factors influence the occurrence and transmission of rabies. It has been observed that the prevalence of rabies in China is positively connected with a number of environmental parameters, including temperature, longitude, distance to country centers, and distance from transportation networks [10]. It is generally accepted that the common vampire bat (*Desmodus rotundus*) is the most important reservoir and vector of rabies in the Western Hemisphere [11,12,13]. Vampire bats typically infect fenced-in cattle while feeding on their blood [14], and their distribution and spread are affected by factors, such as altitude (they favor low elevations) [15], climate change, humidity (high humidity might be required for roosting), season (new births in rainy seasons increase bat movement) [16], and the distance between roosts and interactions with bats’ food sources on farms [17]. A recent study in China found that the rainy season had more human rabies incidences than the dry season [18]. In Mexico, Argentina, and Chile, the distribution of vampire bats is shown to be related to the 10 °C minimum winter isotherm [19]. Since the spatial distribution of reservoir hosts can be altered by climate change and the accompanying rise in temperature, anticipating such shifts is essential for rabies control and eradication strategies [20]. As the ambient temperature goes up, so does the number of reported cases of rabies [21]. Because more animals will be active and able to track greater distances in warmer weather, rabies is more likely to spread [22]. Moreover, as the temperatures rises, people tend to wear less clothing, exposing more flesh, which makes them more of a target for canine attacks [23]. Despite evidence of cattle rabies epidemics linked to vampire bats, which are susceptible to changes in ecological parameters, such as temperature, humidity, and climate [15], the significance of these ecological factors is frequently overlooked [24]. While raccoons, foxes, striped skunks, coyotes, jackals, and yellow mongooses are keeping rabies alive in wildlife, we cannot rule out the possibility of spillover to humans and domestic animals in areas where dog-mediated rabies has been eradicated, such as Europe, the United States, Canada, and Australia [3,25]. Oral rabies vaccinations of wild animals, such as foxes and jackals, have been successful in different countries. Examples include the elimination of sylvatic rabies among foxes in Slovenia [26], efficiency among foxes in Serbia in 2015 [27], and rapidly decreased sylvatic rabies in Croatia until 2014, when the last case was confirmed [28]. Starting from Switzerland in 1987, over the last 4 decades, over 736 million oral rabies vaccine (ORV) baits have been spread across 30 European countries, spanning an area of 2.75 million km^2^ and, for over 50 years, it has been the foundational tool for eradicating the rabies virus from wildlife species all over the world [26,29,30]. Understanding the wildlife ecology, continuous epidemiological studies, and use of ORV can play important role for rabies elimination in sylvatic cycles. 

Understanding street dog ecology and demography [31], as well as waste management, are often overlooked as a control measure in LMICs [32]. Because they influence animal distribution and movement, moderate community access to health services, and the distribution of the human communities at risk, environmental factors, such as distance to healthcare centers, landscape, and weather have been documented as potential risk factors associated with reported cases of rabies [33]. The burden of rabies in LMICs is underestimated, which is attributed to the underreporting of cases of rabies associated with the lack of understanding of ecological perspectives of the occurrence and transmission of rabies [34,35]. When the connection between ecological perspective and land cover types is recognized, it may be possible to obtain a more precise estimate of the disease burden through surveillance operations [10,36]. 

## 3. Socio-Economic Factors

Economic and societal variables contribute to the complexity of the canine rabies crisis [37]. As humans and dogs are housed under the same roof, there is always a risk of rabies until and unless all dogs are properly vaccinated. However, the vaccination of dogs is dependent on the socioeconomic status of the household or the locality. There is a need for rabies education and awareness initiatives including wound treatment (first aid) and PEP, because multiple studies have indicated a knowledge and awareness gap among individuals in LMICs [38,39,40,41,42,43]. Over 65% of Ghanaian respondents to a recent survey held the belief that rabies could be treated with a combination of concoctions, medical herbs, and the eating of offending dog meat [44,45]. The spread of rabies and the effect of dog slaughter, handling, and ingestion are poorly known. Nonetheless, while not being present in meat or blood, exposure to cuts and wounds during slaughter and canine meat processing to saliva or brain tissues may represent a high danger [46]. Dog meat consumption as a delicacy is popular among several tribes in western Africa, particularly in Ghana [47] and Nigeria [48], which increases the risk of people contracting rabies. In a similar vein, research in Nigeria found rabies antigens in the brain of dogs slaughtered for human consumption [46]. The belief of being protected from rabies and evil spirits, the medicinal value, and the taste of its meat all contribute to the consumption of dog meat. Such deeply rooted superstitious beliefs contribute to rabies transmission in those dog meat-eating communities, necessitating significant rabies awareness and education initiatives, as well as pre-exposure prophylaxis (PrEP) and PEP [45,49]. Rabies has been transmitted to humans and has resulted in human mortality in parts of the world where there is a dearth of diagnostic facilities and where superstitious beliefs about animals play a role [35]. There is also the possibility of infection from dog meat due to the exposure of cuts and scrapes during processing to the dog’s saliva and brain [46]. 

Due to socio-cultural norms, laboratory testing of human brain samples is not practical in LMICs; hence, the majority of cases of rabies in humans are identified exclusively based on symptoms. False results, incorrect inference of rabies epidemiology, and associated difficulties in rabies control stem from the lack of laboratory diagnosis [49]. The underreporting of rabies is complicated by the pathophysiology of disease itself. Most individuals with rabies do not bother coming to the hospital for diagnosis since they know it is terminal as soon as the symptoms arise. However, in regions where other diseases with neurological symptoms are common, this leads to rabies being misdiagnosed as other diseases with neurological forms. Because of PEP shortages, a lack of centralized locations where people can get PEP after being bitten, and a lack of oversight over the sale of PEP to private suppliers, it is difficult to keep track of the number of rabies cases diagnosed and the number of people who have been treated for the disease [3,50]. The rural poor, an already vulnerable demographic, are disproportionately affected by these PEP provision issues [10]. In addition, most impoverished countries lack the necessary staff and infrastructure for rabies surveillance and diagnosis; therefore, only a small amount of data of questionable quality is accessible [23,51]. 

The worldwide burden of rabies can only be estimated by extrapolation in the presence of both accurate death reporting systems and a more extensive active surveillance research [52]. While the global burden of rabies should be estimated, an inadequate mortality reporting system and limited active surveillance have been major obstacles. As a means of dealing with this issue, scientists have developed prediction approaches, such as a probabilistic decision tree approach, which may calculate the likelihood that a human will contract clinical rabies after being bitten by a dog suspected of having the virus [53]. Knobel et al. (2005) utilized this technique on the data from different countries to conclude that canine rabies was responsible for about 55,000 deaths per year across Africa and Asia [54]. However, since this publication in 2005, more data has become available, and the dynamics of the disease have shifted, with rising occurrence in some regions and the appearance of rabies in those that had previously been free of it; thus, a new model needs to be developed taking into account the shifting dynamics of rabies in recent years.

The societal costs associated with this fatal disease can be broken down into a number of different categories, including mortality and lost productivity due to premature death, morbidity due to adverse events of vaccination using nerve tissue vaccines, and the psychological effects of exposure to this fatal disease [6]. While the veterinary sector typically incurs costs related to dog vaccination, the medical sector and affected communities bear the direct costs of PEP, depending on the use of rabies immunoglobulin (RIG), and the type of vaccine and regimen, for example, intramuscular (IM) versus intradermal (ID) administration, as well as the indirect costs of seeking PEP (including travel and accommodation for multiple clinic visits and lost income). The veterinary and medical fields are equally responsible for the cost of monitoring. Livestock losses can have a significant negative influence on national and household economies; the extent to which this manifests itself is dependent on the size of the animal population at danger and the degree to which preventative measures are implemented [52,55,56].

Economic factors also play a crucial role in human rabies transmission [10] and PEP [57]. The rabies transmission is linked to the economic growth of a country or a place with a high prevalence of low economic areas [58]. Several previous studies have found that the incidence and transmission of rabies are negatively correlated with economic development [18,58,59]. For example, El Salvador’s rabies control programs were hampered by the economic and social crisis in the country [58]. This poverty results in the deprivation of basic health services, unavailability of PEP and PrEP, and the non-reporting of cases [60]. The capacity for vaccine manufacturing and procurement determines the status of rabies in the country, which in turn depends on the economic well-being of the country [32,61]. In developing countries, the cost of post-exposure rabies is so high that it is beyond the affordability of the majority of its citizen. For example, in Ethiopia, the average cost of full-dose vaccination (PEP) is 170 US dollars [62], while per capita gross national income is $890 [63]. The dose-sparing intradermal (ID) regimens that help to reduce the cost of the post-exposure vaccine [64] are available in the market, but these options are also not easily accessible and affordable to much of the population in LMICs. Furthermore, when a human is bitten by a rabid animal, the best PEP measure is to use RIG; however, RIG availability in LMICs is very limited, and the majority of patients in rural areas are out of reach of such RIG facilities; thus, economically weak patients have to succumb to death. Therefore, the economic aspect is critical in rabies control and elimination. Apart from all of these socioeconomic concerns, vaccine quality is also a matter of concern. The quality and effectiveness of the rabies vaccines available in the market should be tested, and only high-quality vaccines should be made available to the customers. We believe that while developing and conducting rabies control programs, the ecological and socioeconomic variables of a specific location should be taken into account. Unfortunately, the majority of rabies control programs seem to have ignored these factors, which could be the reason for the lack of intended success in rabies control programs.

The cost of dog vaccinations, the only scientifically established method of limiting human exposures and stopping the spread of the disease, is negligible [65,66]. The United States of America (USA) is one country that has maintained a significant investment in dog vaccination ($0.11/person/year) [67,68]. As a result, the cost of preventing rabies in the USA is relatively low ($0.02/person/year), and the rabies burden is also low (200 deaths per year across the continent, largely in Haiti) compared to regions where dog vaccination is not widely practiced [54]. As opposed to the internationally coordinated effort in Latin America, many developing countries have left rabies control and prevention to the private sector with no legal obligations or incentives (for example, as part of structural adjustment programs in sub-Saharan Africa). Therefore, rabies has been ignored, since it is not as financially significant as other livestock diseases [55,69]. Even while veterinary expenses were generally lower, investments in canine vaccinations had the potential to reduce medical expenditures, highlighting the importance of intersectoral coordination. Despite the World Bank’s recognition of zoonoses prevention and control as a “public good” and its backing for efforts to improve veterinary services (e.g., via the OIE Performance of Veterinary Services pathway), these efforts have been hampered by a lack of funding [69,70]. Vaccine prices vary widely from country to country, making it difficult for developing nations to execute mass vaccination programs without assistance from wealthier nations [68,71]. Potentially crucial roles could be played by vaccine banks, such as those managed by OIE [61,66].

Public education is crucial in order to influence the behavior of owners towards rabies prevention. It is imperative that relevant authorities consistently and systematically educate the public on how to prevent and manage rabies in their communities [6,72]. Most importantly, in LMICs, residents should be educated on the need to limit dog ownership and transportation, the need to vaccinate young dogs, and the importance of controlling reproduction [10,73]. Though rabies is 100% preventable, it still places a heavy strain on the world’s population [3,25]. Investment in rabies control, which has been woefully deficient, is crucial to solving the problem. Elimination is possible with currently employed methods, and long-term mass vaccination programs for dogs could lower medical sector and societal expenses; however, creative financing approaches are necessary to overcome institutional impediments.

The canine population can be successfully immunized either with a subcutaneous vaccination or through ORV baits. However, for such immunization, national governments and local health authorities should commit to making rabies control a priority and keeping the infrastructure in place to regularly vaccinate the dog population against the disease. A deeper familiarity with the ecology and population dynamics of local dog populations can only aid vaccination drives. In the absence of this information, there is a risk of losing or squandering resources. As a corollary, it is important that dog owners in developing nations have access to and can afford veterinary care in order to keep canine populations stable.

## 4. Conclusions

Future research on dog ecology should concentrate on the nations with the highest endemicity of canine rabies. The rapid churn in the dog population must be slowed, and new methods of increasing canine longevity must be investigated. Eliminating rabies in both dogs and humans will need widespread implementation of programs that foster humane dog management and advocate for ethical pet ownership. The World Health Organization (WHO), the Food and Agriculture Organization (FAO), the World Organization for Animal Health (OIE), and the Global Alliance for Rabies Control (GARC) all agree on The Global Strategic Plan [74] to end deaths from dog-mediated human rabies by 2030. However, without considering ecological and socioeconomic factors which are often neglected, the success of the above-mentioned plan is slight and hardly realistic.

## Data Availability

Not applicable.
